# Epicardial Adipose Tissue and Cardiovascular Risk Assessment in Ultra-Marathon Runners: A Pilot Study

**DOI:** 10.3390/ijerph18063136

**Published:** 2021-03-18

**Authors:** Michał Konwerski, Marek Postuła, Marzena Barczuk-Falęcka, Anna Czajkowska, Anna Mróz, Katarzyna Witek, Wawrzyniec Bakalarski, Aleksandra Gąsecka, Łukasz A. Małek, Tomasz Mazurek

**Affiliations:** 1 1st Chair and Department of Cardiology, Medical University of Warsaw, 02-097 Warsaw, Poland; konwerski.mich@gmail.com (M.K.); aleksandra.gasecka@wum.edu.pl (A.G.); 2 Center for Preclinical Research and Technology CEPT, Department of Experimental and Clinical Pharmacology, Medical University of Warsaw, 02-097 Warsaw, Poland; m.postula@wum.edu.pl; 3 Department of Pediatric Radiology, Medical University of Warsaw, 02-091 Warsaw, Poland; marzena.barczuk-falecka@wum.edu.pl; 4 Faculty of Tourism and Recreation, Józef Piłsudski University of Physical Education in Warsaw, 00-968 Warsaw, Poland; anna.czajkowska@awf.edu.pl; 5 Faculty of Physical Education, Józef Piłsudski University of Physical Education in Warsaw, 00-968 Warsaw, Poland; anna.mroz@awf.edu.pl (A.M.); katarzyna.witek@awf.edu.pl (K.W.); wawbak@gmail.com (W.B.); 6 Department of Epidemiology, Cardiovascular Disease Prevention and Health Promotion Institute of Cardiology, 04-628 Warsaw, Poland; lukasz.a.malek@gmail.com

**Keywords:** epicardial adipose tissue, ultrarunners, inflammation, cardiac magnetic resonance, cardiovascular disease

## Abstract

Epicardial adipose tissue (EAT) volume is associated with cardiovascular disease (CVD). Data regarding the influence of extremely intensive training on CVD are scarce. We compared EAT volume among ultra-marathon runners and in the sedentary control group, and assessed the correlations between EAT and risk factors of coronary artery disease (CAD). EAT volume around three main coronary vessels and right ventricle (RV) was measured in 30 healthy amateur ultrarunners and 9 sex- and age-matched sedentary controls using cardiac magnetic resonance. In addition, body composition, lipid profile, interleukin-6 (IL-6) plasma concentration, and intima-media thickness (IMT) were measured as well. The EAT volume was lower in all measured locations in the ultrarunners’ group compared to control group (*p* < 0.001 for all). Ultrarunners had lower BMI and fat percentage (FAT%) and more favorable lipid profile compared to the control group (*p* < 0.05 for all). Ultrarunners had lower rate of pathologically high levels of plasma IL-6 (>1 pg/mL) compared to the control group (17% vs. 56%, *p* < 0.05). IMT was similar in both groups. In the ultrarunners’ group, there was a positive correlation between EAT surrounding left anterior descending artery, circumflex artery, and RV and FAT%, and between EAT around circumflex artery and LDL and non-HDL cholesterol (*p* < 0.05 for all). In summary, extremely intensive training may decrease the risk of cardiovascular events in adult population of amateur athletes by reducing the amount and pro-inflammatory activity of EAT. However, more research is needed to draw firm conclusions regarding the anti- and pro-inflammatory effects of intensive training.

## 1. Introduction

Despite the constant progress in pharmacological and interventional treatment, the incidence of cardiovascular diseases (CVDs) is still growing. In 2012 and 2013, CVDs were responsible for 17.3 million deaths worldwide, which makes them the leading cause of mortality [1,2,3]. A number of risk factors for CVDs have already been described and many of them are potentially reversible. In the worldwide INTERHEART study, there were nine potentially modifiable factors in patients with myocardial infarction: smoking, dyslipidemia, arterial hypertension, diabetes mellitus, abdominal obesity, psychosocial factors, daily consumption of fruits and vegetables, regular alcohol consumption, and regular physical activity [4].

Obesity and physical activity are potentially easily reversible risk factors. Particularly, the abdominal type of obesity is one of the most important factors predisposing to atherosclerosis and cardiovascular events [5,6]. The volume and thickness of epicardial adipose tissue (EAT) was showed to correlate with intra-abdominal fat mass, severity of obesity [7,8], and the incidence of cardiovascular events [9]. EAT is a visceral fat with high metabolic activity, located between the pericardium and the myocardium and not separated from myocardium and coronary vessels [10,11]. EAT activity solely depends on the metabolic state. In patients with metabolic syndrome, perivascular fat surrounding coronary arteries (pericoronary adipose tissue, PCAT), which is a subtype of EAT, loses its protective capability and becomes an aggressive, pro-inflammatory tissue, releasing cytokines and chemokines. Recently, it was showed that the carotid intima-media thickness (IMT), which is a very strong indicator of systemic atherosclerosis, was associated with EAT thickness measured by echocardiography, independently from body mass index (BMI) and waist circumference (WC) [11]. Hence, PCAT may be directly involved in the pathogenesis of coronary artery disease (CAD) [12,13,14].

Moderate physical activity has a protective effect against CAD and decreases all-cause mortality [4,15,16,17,18,19]. This effect can be caused by increasing HDL concentration in serum, lowering blood pressure, weight reduction, and a reduction in insulin resistance. Recently, the number of people practicing sports in developed countries has been growing geometrically. Especially, endurance running has gained popularity and probably is the most popular sport worldwide in middle-age adults [17].

Since little is known about the influence of extreme high-intensity training on cardiovascular risk factors, we sought to compare EAT volume assessed with cardiac magnetic resonance (CMR) among ultra-marathon runners and in the sedentary control group, and assess the correlations between EAT and risk factors of CAD (body composition, venous blood lipid profile, interleukin-6 (IL-6) plasma concentration, and IMT).

## 2. Materials and Methods

**Figure 1:**
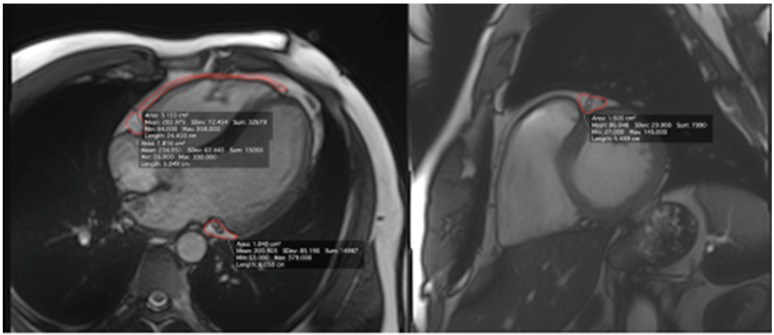
Example of pericoronary adipose tissue calculation with cardiac magnetic resonance by measurement of the area around the main coronary arteries and over the free wall of the right ventricle (epicardial adipose tissue).

The study included a group of 30 healthy, male, amateur experienced ultra-marathon runners and 9 gender- and age-matched sedentary controls [20]. All study participants underwent CMR with 3T scanner (Siemens, Erlangen, Germany) in which the EAT area was measured in 4 points: (1) in the vicinity of the free right ventricle (RV) wall, (2) surrounding right coronary artery (RCA) and (3) circumflex artery (Cx) on 4-chamber cine balanced steady-state free processing (b-SSFP) images, and (4) in the neighborhood of the left anterior descending (LAD) coronary artery on the basal short axis b-SSFP image. Figure 1 shows an example of PCAT calculation with CMR by measurement of the area around the main coronary arteries and over the free wall of the RV.

Body composition was analyzed by Tanita body composition analyzer (Tanita Europe BV, Amsterdam, The Netherlands). Venous blood lipid profile was measured at fasting in the accredited hospital laboratory. Interleukin-6 (IL-6) plasma concentration were analyzed using the commercially available enzyme-linked immunosorbent assay (ELISA; Roche Diagnostics, Indianapolis, IN, USA). Intima-media thickness (IMT) was measured using carotid ultrasonography (Siemens ACUSON S1000 Ultrasound System, HELX Evolution with Touch Control). IMT was determined by B-mode ultrasound with a 5–14 MHz linear transducer. The measurements were performed using sagittal imaging of the common carotid artery (CCA). At least five measurements on each side were taken to obtain an average value. IMT was assessed on the posterior wall of the CCA on the right and on the left, 1 cm from the carotid bifurcation. According to the European Society of Cardiology (ESC) Cardiovascular Disease (CVD) Prevention in Clinical Practice Guidelines 2016, we set the cut-off point to values > 0.9 mm [21].

## 3. Results

Description of ultra-marathon runners is shown in Table 1.

The participants’ baseline characteristics and results are shown in Table 2.

The EAT volume was smaller in all localizations in the ultra-marathon runners as compared to control group (*p* <0.001 for all). Regarding body composition, the control group had higher mean body weight, BMI, and fat mass (FAT) (*p* < 0.001) as compared to ultra-marathon runners. The concentrations of total cholesterol, low-density lipoprotein (LDL) cholesterol, non-high-density lipoprotein (non- HDL) cholesterol, and triglycerides were higher, and the concentration of high-density lipoprotein (HDL) cholesterol was lower in the control group compared to ultra-marathon runners (*p* < 0.05 for all parameters). There were no differences in the IL-6 plasma concentration between the groups (*p* = 0.16). However, in the runner’s group, the rate of pathologically high levels of plasma IL-6 (>1 pg/mL) was 3-fold lower compared to the control group (17% vs. 56%; *p* < 0.05). IMT was similar in both groups.

**Figure 2:**
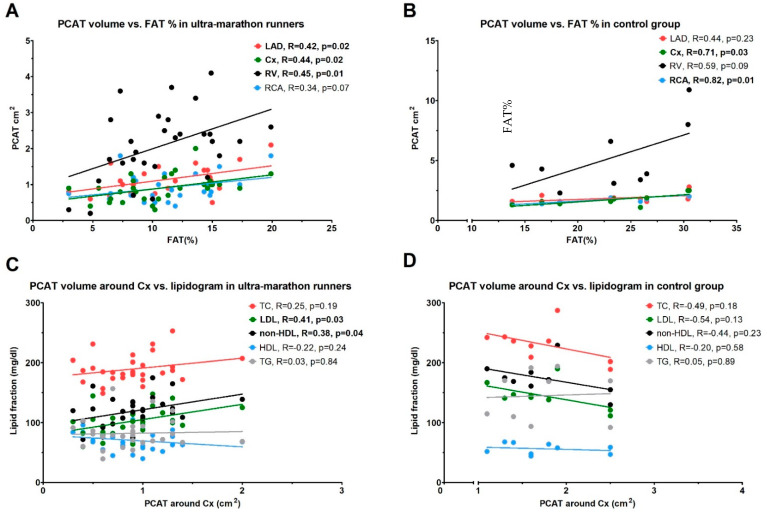
(**A**,**B**) Correlations between body fat percentage (FAT%) and pericoronary adipose tissue (PCAT) in the ultra-marathon runners and control group. (**C**,**D**) Correlations between PCAT around the circumflex artery (Cx) and lipidogram parameters. LAD—left anterior descending artery; RV—right ventricle; RCA—right coronary artery; LDL—low-density lipoproteins; HDL—high-density lipoproteins; non-HDL—non-high-density lipoproteins; TC—total cholesterol; TG—triglycerides.

Figure 2 shows the correlations between body FAT% and PCAT, and between PCAT around the Cx artery and lipidogram parameters in the ultra-marathon runners and control group.

There was an intermediate, positive correlation between FAT% and PCAT around LAD, Cx, and RV in the runners’ group (R ≥ 0.42 for all; *p* ≤ 0.02 for all; Figure 2A) and a strong, positive correlation between FAT% and PCAT around Cx and RCA in the control group (R ≥ 0.71; *p* ≤ 0.03 for both; Figure 2B). Moreover, there was an intermediate position correlation between the PCAT around Cx artery and the concentration of LDL and non-HDL in the runners’ group (R ≥ 0.41; *p* ≤ 0.03 for both; Figure 2C), which was not observed in the control group (group (Figure 2D). There were no significant correlations between PCAT and FAT% with IL-6 plasma concentrations and IMT in both studied groups.

## 4. Discussion

The purpose of this study was to compare the EAT volume assessed with CMR among ultra-marathon runners and in the sedentary control group, and assess the correlations between EAT and risk factors of CAD, including body composition, venous blood lipid profile, IL-6 plasma concentration, and IMT. The main findings of this study are that (i) EAT volume is smaller in all measured localizations in the ultra-marathon runners compared to the control group, and (ii) there is a positive correlation between FAT% and PCAT surrounding LAD artery, Cx artery, and RV in the ultra-marathon runners, and a positive correlation between LDL and non-HDL concentration and PCAT around Cx artery.

CVD and obesity are significant health problems in developed and developing countries [1,2,3,5,6]. Strong connections between obesity, insulin resistance, and atherogenic lipids profile are well-documented [22]. Whereas subcutaneous adipose tissue (SAT) is not associated with cardiovascular risk, visceral fat seems to play a major role in atherosclerosis [23]. Recently, there is a growing interest in PCAT, which is a specific visceral tissue located in the direct neighborhood of the coronary arteries and therefore, in addition to the systemic effect, it also has paracrine and vasocrine effect on the surrounding vessels [10,24,25]. There are many differences at the anatomic, histologic, embryologic, and biomolecular levels between EAT/ PCAT and other fat depots [12,13]. Inflammatory processes underlying atherosclerosis are well-documented [26]. In addition, EAT was showed to participate in the pathogenesis of coronary atherosclerosis by secreting multiple cytokines and chemokines, thus serving as an active pro-inflammatory tissue [14,27]. In patients with advanced CAD undergoing coronary artery bypass grafting, EAT released more inflammatory markers than the SAT [27]. Moreover, the thickness of EAT measured using computed tomography reflected the stage of CAD. There was also a positive correlation between PCAT and vulnerability of atherosclerotic plaques based on their composition, assessed by intravascular ultrasound imaging [28]. Mazurek et al. analyzed the composition of atherosclerotic plaques using virtual histology intravascular ultrasonography and quantitatively assessed PCAT using positron emission tomography/computed tomography among patients with acute coronary syndrome without persistent ST-segment elevation. The inflammatory activity was measured by maximal standardized uptake value of 18-fluorodeoxyglucose. Inflammatory activity within PCAT was higher than in subcutaneous, visceral thoracic or epicardial adipose tissue, and there was a strong, positive correlation with pro-inflammatory activity of PCAT and both coronary plaque burden and its necrotic core component [29]. The association between EAT, atrial fibrillation [30], and impairment of cardiac function was also demonstrated [31]. Recently, it was demonstrated that among patients without known cardiovascular disease, the mean EAT thickness evaluated in magnetic resonance was higher in those with subclinical left ventricle impairment compared to those with normal left ventricle function, independently of traditional risk factors [32]. It has been also shown that patients with a familiar hypercholesterolemia had increased EAT thickness, and there was a positive correlation between EAT and plasma LDL concentration in these patients [33]. Volume and thickness of EAT correlates with intra-abdominal fat mass, obesity extent [7,8], and is independently associated with cardiovascular events [9]. Further, EAT is an independent predictor for the presence of carotid plaque [11]. It has been emphasized that EAT has a stronger association with systemic inflammation and subclinical atherosclerosis (IMT than BMI and waist circumference) [34,35].

The effect of very intense physical activity on physiological processes is still unknown. Both short-term and long-term endurance sport can induce both anti- and pro-inflammatory response. Long-term physical exercise was shown to reduce the level of C-reactive protein (CRP), especially when accompanied by a decrease in weight, especially in individuals with initially elevated CRP [36]. On the other hand, long-term exercise may also stimulate inflammatory processes, vascular oxidative stress, and fatigue of the elastic components of the vessel wall [37]. For example, obese non-elite runners had higher CRP plasma concentration than lean elite runners, measured 24 h after marathon. In addition, obese non-elite runners had a higher level of IL-6 and a lower level of IL-10 at baseline than lean elite and lean non-elite runners [38]. These results indicate the association between adipose tissue and inflammation.

In our population of extreme amateur runners, EAT volume was smaller in all measured locations compared to control group. As expected, runners had lower weight, BMI, fat free mass, body FAT%, concentrations of LDL and TG, higher concentration of HDL, and less frequently pathologically high levels of plasma IL-6 compared to controls. These findings suggest that high intensity training may have a favorable effect on cardiovascular risk factors. On the other hand, there was a correlation between EAT/PCAT surrounding LAD artery, Cx artery, and RV, and between PCAT surrounding Cx artery and lipid profile in the ultra-marathon runners’ group, which might potentially reflect increased cardiovascular risk in this population.

## 5. Limitations

The main limitation of this study is the small sample size. For example, likely the correlations between FAT% and PCAT around coronary arteries, which were not significant in our control group, would become significant in a larger cohort. The second limitations are the limited inclusion criteria of the study population, including homogenous individuals regarding gender (only male) and ethnicity (only Caucasian, Polish). Therefore, our results are not applicable to female and to any other ethnic group. Another limitation is the control group comprising sex- and age-matched sedentary controls with higher body weight, BMI, and FAT levels, which are known risk factors of CVD. Ideally, the control group would comprise individuals with moderate physical activity and similar body composition to the study group. However, such group was not available when we performed this study. Finally, the correlations showed in our study do not prove any causal relationship between intensive training, EAT, and CVD. Due to high risk of bias in this study, we may only speculate about the benefit and risk of extremely intensive training on cardiovascular health, and further research is required to ultimately answer this question.

## 6. Conclusions

In summary, extremely intensive training may decrease the risk of cardiovascular events in adult population of amateur athletes by reducing the amount and pro-inflammatory activity of EAT. More research is needed to draw firm conclusions regarding the anti- and pro-inflammatory effects of intensive training.

## Figures and Tables

**Table 1:** Description of ultra-marathon runners. IQR—interquartile range.

Parameter	Ultra-Marathon Runners *n* = 30, Median (IQR)
Years of running	9 (7–15)
Age at start of ultra-running	34 (29–39)
Total covered distance (km)	25,000 (20,000-40,000)
Weekly running distance (km)	80 (70–90)
Number of ultra-races completed	15 (10–27.5)
Number of ultra-races during last 2 years	5.5 (4–9)
Number of completed ultra-races >100 km	3.5 (2–7)
Best place achieved in an ultra-race	5 (1–13)

**Table 2:** Participants’ baseline characteristics and results.

**Baseline Characteristics**			
**Parameter**	**Ultra-marathon Runners (*n* = 30)**	**Control Group (*n* = 9)**	***p***
**Age (years)**	40.93 ± 6.57	40.78 ± 8.32	0.95
**Heigth (cm)**	172.02 ± 32.49	179.89 ± 6.43	0.48
**Weight (kg)**	72.73 ± 5.19	96.10 ± 19.39	<0.001
**BMI (cm/m^2^)**	23.09 ± 1.54	30.00 ± 5.41	<0.001
**FAT % (%)**	10.78 ± 4.01	23.17 ± 5.91	<0.001
**FAT mass (kg)**	8.02 ± 3.32	23.16 ± 10.16	<0.001
**FFM (kg)**	65.30 ± 3.44	74.34 ± 14.86	0.004
**TBW (kg)**	47.81 ± 2.52	53.40 ± 7.17	<0.001
**Blood Test Results**			
**Parameter**	**Ultra-marathon Runners (*n* = 30)**	**Control Group (*n* = 9)**	***p***
**TC (mg/dl)**	189.66 ± 23.38	230.22 ± 28.60	<0.001
**HDL (mg/dl)**	70.52 ± 15.96	56.33 ± 8.99	0.016
**LDL (mg/dl)**	102.68 ± 22.45	144.86 ± 23.12	<0.001
**non-HDL (mg/dl)**	119.14 ± 25.07	173.89 ± 27.10	<0.001
**TG (mg/dl)**	82.27 ± 24.73	145.07 ± 41.82	<0.001
**IL-6 (pg/mL)**	1.29 ± 0.75	1.70 ± 0.72	0.156
**Results of Imaging Tests**			
**Cardiac Magnetic Resonance**			
**Parameter**	**Ultra-marathon Runners (*n* = 30)**	**Control Group (*n* = 9)**	***p***
**LAD (cm^2^)**	1.12 ± 0.4	1.86 ± 0.41	<0.001
**RCA (cm^2^)**	0.88 ± 0.39	1.78 ± 0.34	<0.001
**Cx (cm^2^)**	0.90 ± 0.36	1.74 ± 0.49	<0.001
**RV (cm^2^)**	2.07 ± 0.97	5.23 ± 2.77	<0.001
**Ultrasonography**			
**Left IMT (cm)**	0.07 ± 0.02	0.07 ± 0.01	0.99
**Right IMT (cm)**	0.08 ± 0.03	0.08 ± 0.01	0.68

FAT%—body fat percentage, BMI—body mass index, FFM—fat free mass, TBW—total body water, TC—total cholesterol concentration; HDL—high-density lipoprotein concentration; LDL—low-density lipoprotein concentration; non-HDL—non-high-density lipoprotein concentration; TG—triglyceride concentration; IL-6—interleukin-6 concentration; LAD—PCAT area around left anterior descending artery; RCA—PCAT area around right coronary artery; Cx—PCAT area around circumflex artery; RV—EAT area over right ventricle; IMT—carotid intima-media thickness.

## Data Availability

The data presented in this study are available on request from the corresponding author. The data are not publicly available due to patients’ privacy.
